# The efficacy and physiological bases of small muscle mass exercise in health and disease

**DOI:** 10.1113/EP093247

**Published:** 2026-02-05

**Authors:** Callum G. Brownstein

**Affiliations:** ^1^ School of Biomedical, Nutritional, and Sport Sciences, Faculty of Medical Sciences Newcastle University Newcastle upon Tyne UK

**Keywords:** angiogenesis, fatigability, large muscle mass exercise, mitochondria, small muscle mass exercise, vascular blood flow

## Abstract

The conventional approach to aerobic exercise prescription involves large muscle mass exercise and the manipulation of variables such as training intensity, duration and frequency to promote desired adaptations. However, during whole‐body exercise, central limitations (i.e., neural, pulmonary and/or cardiac) constrain exercise tolerance and limit the increase in muscle blood flow and the degree of intramuscular metabolic perturbation incurred. Consequently, even during high‐intensity large muscle mass exercise, a substantial peripheral reserve remains, potentially diminishing the adaptive stimuli that drive improvements in peripheral function and, in turn, exercise tolerance. In contrast, these central constraints are markedly attenuated during small muscle mass aerobic exercise, such as single‐leg cycling or knee extension. As a result, muscle activation, blood flow, work rate and the magnitude of metabolic perturbation per unit of muscle are considerably greater during small compared with large muscle mass exercise. Because many of these responses are thought to represent key triggers initiating peripheral adaptations, such as angiogenesis and mitochondrial biogenesis, small muscle mass exercise might confer unique advantages for enhancing peripheral vascular and metabolic function. This review outlines the key physiological differences between small and large muscle mass exercise, their relevance to peripheral adaptations, and current evidence on the efficacy of small muscle mass exercise in improving peripheral function and exercise tolerance in performance, health and disease.

## Abstract (highlights)

- **What is the topic of this review?**
  This review focuses on key differences in the physiological responses between small and large muscle mass exercise and their potential relevance in the adaptive response to chronic exercise exposure.
- **What advances does it highlight?**
  The review proposes that the augmented local blood flow and metabolic perturbation during small muscle mass exercise could provide a potent stimulus eliciting improvements in peripheral vascular and metabolic function in performance, health and disease.

## 1 INTRODUCTION

Chronic exposure to aerobic exercise is associated with a myriad of physiological adaptations that promote improvements in exercise tolerance and health (Granata et al., 2018; Green et al., 2017; Hellsten & Nyberg, 2015). Although the mechanisms by which exercise elicits these improvements are incompletely understood, current theory posits that the acute metabolic and mechanical stressors associated with exercise activate molecular signalling pathways, leading to an increase in the content and/or function of specific proteins that ultimately attenuate cellular perturbations in response to subsequent bouts of physical activity (Coffey & Hawley, 2007). To elicit these stressors, exercise training variables, such as intensity, duration and frequency, are manipulated with the aim of promoting desired adaptations and improvements in performance. Accordingly, there is great interest, both in the scientific literature and in applied practice, in understanding how training variables can best be prescribed to optimize physiological adaptations and, in turn, enhance performance and exercise tolerance.

Two key peripheral physiological adaptations to aerobic exercise training are improvements in capillary density and mitochondrial content. The importance of these adaptations stems from the limitations that capillaries and mitochondria can impose on oxygen extraction and cellular bioenergetics. Specifically, although the single cell layer composition and vast surface area provided by capillaries make them ideally suited to mediate diffusive oxygen transport, oxygen extraction nevertheless remains incomplete during maximal exercise and when a metabolic reserve is present, suggesting that diffusive limitations at the capillary–myocyte interface limit maximum oxygen consumption (${\dot{V}}_{O_{2}\max}$) (Wagner, 1996). Consequently, the increases in capillary density associated with aerobic exercise training facilitate improvements in muscle oxygen diffusion capacity (Esposito et al., 2018). Although mitochondrial oxidative capacity is not thought to limit ${\dot{V}}_{O_{2}\max}$ in healthy, active individuals (Gifford et al., 2016), oxidative capacity is nevertheless crucial in sustaining energy supply through oxidative phosphorylation, attenuating the intracellular disturbances that impair contractile function and reducing reliance on finite carbohydrate stores (Gollnick & Saltin, 1982), all of which are improved with training (Green et al., 2012; Leblanc et al., 2004; Phillips et al., 1996). The importance of these peripheral factors is underscored by their close correlation with key indices of exercise tolerance, such as the lactate threshold, critical power and ${\dot{V}}_{O_{2}\max}$ (McDougall et al., 2023; Mitchell et al., 2018; Saltin et al., 1986).

The conventional approach when prescribing aerobic exercise training with the aim of promoting increases in capillary density and mitochondrial content is to use large muscle mass exercise, such as running or cycling. However, one limitation of implementing large muscle mass exercise with the aim of inducing peripheral adaptations is that central constraints (i.e. neural, pulmonary and/or cardiac) limit exercise tolerance and inhibit the increase in mass‐specific blood flow and local metabolic stress (Davies & Sargeant, 1975; Zhang et al., 2021), both of which represent key signals in initiating peripheral adaptation (Bishop et al., 2019a; Egginton, 2009). Accordingly, these central limitations, which are particularly prominent in individuals with cardiopulmonary dysfunction, might thereby restrict the adaptive stimuli associated with improvements in peripheral function and, in turn, compromise improvements in exercise tolerance.

It is well established that, during small muscle mass exercise, such as single‐leg knee extension or single‐leg cycling, mass‐specific work rate, muscle activation, blood flow and the degree of metabolic stress incurred are greater than during large muscle mass exercise (Andersen & Saltin, 1985; Cardinale et al., 2019; Mortensen et al., 2005; Richardson et al., 1993; Zhang et al., 2025). Higher mass‐specific blood flow was first demonstrated through the work of Bengt Saltin using single‐leg dynamic knee extension (Andersen & Saltin, 1985), and augmented metabolic stress is inferred from the substantially greater contractile impairment at task failure during small than large muscle mass exercise (Rossman et al., 2012; Zhang et al., 2021). Given that the metabolic perturbations incurred during exercise are key in initiating transcriptional factors that promote adaptation (Fiorenza et al., 2018), small muscle mass aerobic exercise could confer unique advantages for enhancing peripheral vascular and metabolic function whilst mitigating the central constraints associated with large muscle mass exercise. Studies in both healthy and clinical populations provide support for the efficacy of small muscle mass exercise training in eliciting improvements in peripheral function (Abbiss et al., 2011; Broxterman et al., 2021; Esposito et al., 2018; Skattebo et al., 2020b; Tyni‐Lenné et al., 1999).

The present review initially outlines the importance of capillary density and mitochondrial content for exercise tolerance, in addition to the primary mechanisms through which exercise promotes increases in these variables. Establishing this physiological foundation clarifies why the magnitude of local haemodynamic and metabolic stress is integral in promoting adaptation. This, in turn, provides context for the subsequent sections, which examine the key physiological differences between large and small muscle mass exercise of locomotor muscles (e.g., cycling vs. single‐leg knee extension) and the implications of these differences in promoting peripheral adaptations. Finally, evidence supporting the efficacy of small muscle mass exercise and directions for future research are addressed.

## 2 IMPORTANCE OF CAPILLARY AND MITOCHONDRIAL DENSITY FOR EXERCISE TOLERANCE

### 2.1 Capillary density

Capillary density is associated with enhanced muscle oxygen diffusion capacity ($D_{mO_{2}}$), which, based on Fick's law $\left[{\dot{V}}_{O_{2}}=D_{mO_{2}}\times \left(\mathrm{capillary}P_{O_{2}}-\mathrm{mitochondrial}P_{O_{2}}\right)\right]$, permits a greater oxygen extraction and ${\dot{V}}_{O_{2}}$ at any given capillary oxygen pressure (Mortensen et al., 2017; Pilotto et al., 2022). Specifically, by increasing the number of red blood cells adjacent to the respiring myocyte at any given moment, which represents a key determinant of $D_{mO_{2}}$, higher capillary density increases the surface area available for gaseous exchange (Pilotto et al., 2022; Poole & Musch, 2023; Poole et al., 2022). Indeed, endurance exercise training results in parallel increases in both capillary density and oxygen extraction (Kiens et al., 1993). However, given that capillary density is associated with mitochondrial volume (Hoppeler et al., 1985; Ingjer, 1979), which is also associated with oxygen extraction (Cardinale et al., 2019), and because exercise training results in a myriad of central and peripheral adaptations alongside increases in capillary density, direct evidence on the isolated effects of capillary density on diffusive conditions for oxygen is scarce. However, recent work by Pilotto et al. (2022) found that capillary density was associated with near‐infrared spectroscopy‐derived estimates of $D_{mO_{2}}$, determined by assessing muscle ${\dot{V}}_{O_{2}}$ recovery kinetics in the presence of low tissue oxygen saturation, during which ${\dot{V}}_{O_{2}}$ is heavily dependent on $D_{mO_{2}}$. Moreover, using a chronic period (4 weeks) of pharmacologically elevated basal shear stress, which represents a key signal inducing capillary growth (angiogenesis), Mortensen et al. (2017) were able to isolate the effects of increased capillary density on oxygen extraction. Specifically, chronically elevated basal shear stress resulted in a 24% increase in capillary density, concurrent with a 4%–5% increase in oxygen extraction and a 6%–7% reduction in leg blood flow during moderate‐intensity knee‐extensor exercise, with unchanged submaximal ${\dot{V}}_{O_{2}}$. The finding that these improvements occurred in the absence of mitochondrial adaptations highlights the important role of capillary density in oxygen extraction. Accordingly, the higher $D_{mO_{2}}$ in endurance‐trained individuals plays an important role in contributing to higher oxygen extraction and ${\dot{V}}_{O_{2}\max}$ and to a reduced blood flow for a given ${\dot{V}}_{O_{2}}$ at submaximal workloads (Kiens et al., 1993; Skattebo et al., 2020a).

In addition to enhanced oxygen extraction, the improved conditions for oxygen diffusion with higher capillary density are likely to have a positive downstream influence on metabolic control (Hughson et al., 2001; Iaia et al., 2011). For example, in the aforementioned study, Mortensen et al. (2017) found 6%–7% lower femoral lactate levels during submaximal knee‐extensor exercise at the same submaximal ${\dot{V}}_{O_{2}}$. Moreover, short‐term training has been shown to improve metabolic stability (i.e., attenuate exercise‐induced alterations in ADP, PCr and P_i_) concurrent with increases in capillary density and prior to improvements in oxidative capacity taking place (Green et al., 2012; Layec et al., 2013). These studies provide evidence for an independent role of capillary density in improving metabolic stability.

The positive association between capillary density and metabolic stability could be explained by the higher intramuscular $P_{O_{2}}$ afforded through an enhanced capillary density and $D_{mO_{2}}$ (Hughson et al., 2001). Specifically, intracellular $P_{O_{2}}$ represents an important modulator of cellular bioenergetics, with higher $P_{O_{2}}$ being associated with a smaller rise in NADH/NAD^+^ and a smaller decrease in phosphorylative potential (ATP/ADP × P_i_) during muscle contractions (Hogan et al., 1992, 1998; Katz & Sahlin, 1987; Wilson & Erecińska, 1985). In turn, decreasing ATP/ADP × P_i_ represents a powerful stimulus for glycolytic activation through the allosteric effects of ADP on key regulatory points in glycolysis, and an increase in NADH/NAD^+^ stimulates the reduction of pyruvate to lactate (Clanton et al., 2013; Katz & Sahlin, 1990). This enhanced metabolic stability is likely to contribute to the association between capillary density and critical power (Mitchell et al., 2018) and endurance exercise performance (Coyle et al., 1988; Iaia et al., 2011).

### 2.2 Mitochondrial content

Increases in mitochondrial content and associated increases in the concentration of mitochondrial enzymes accelerate the maximal rate of oxidative ATP generation (i.e., mitochondrial respiratory capacity, *V*_max_). Although this respiratory capacity is well in excess of metabolic demand in trained individuals, even at ${\dot{V}}_{O_{2}\max}$ (Boushel et al., 2011; Gifford et al., 2016), increases in *V*_max_ play a pivotal role in improving the sensitivity of respiratory control (Gollnick & Saltin, 1982). Specifically, an increased mitochondrial content is associated with a smaller relative change in the putative modulators of oxidative phosphorylation (ATP, ADP, P_i_, PCr and Cr; Balaban, 1990; Chance & Williams, 1955; Dudley et al., 1987; Wilson, 1994). This has been demonstrated across several studies displaying attenuated alterations in PCr, P_i_ and ADP at a given work rate and ${\dot{V}}_{O_{2}}$, concurrent with increases in mitochondrial content following a period of endurance training (Green et al., 1995a, 1995b; Leblanc et al., 2004; Phillips et al., 1996).

There are several beneficial consequences to the improved metabolic stability associated with increased mitochondrial content. First, the attenuated increase in ADP, P_i_ and AMP reduces the allosteric activation of key regulatory glycolytic enzymes, thereby reducing glycolytic flux, carbohydrate oxidation and lactate formation at a given submaximal work rate (Green et al., 1995b). In turn, the lower reliance on carbohydrate oxidation, coupled with an increase in the concentration of mitochondrial enzymes responsible for β‐oxidation (Molé et al., 1971), permits a greater production of acetyl‐CoA from fatty acids. The greater contribution of fatty acid oxidation helps to spare finite stores of muscle glycogen, the depletion of which is associated with impairments in excitation–contraction coupling (Ørtenblad et al., 2013), adenine nucleotide loss (Norman et al., 1988) and endurance performance (Bergström et al., 1967). Second, given that metabolites, such as P_i_, have potent inhibitory effects on excitation–contraction coupling and myofibrillar force production (Allen & Trajanovska, 2012; Sundberg & Fitts, 2019), greater respiratory sensitivity is likely to contribute to an attenuation of neuromuscular fatigability at a given work rate (Bachasson et al., 2016; Ducrocq et al., 2021; McDougall et al., 2023). Third, the improvement in metabolic stability with higher mitochondrial content means that the critical levels of metabolic instability associated with critical power are attained at a higher power output and ${\dot{V}}_{O_{2}}$ (Goulding & Marwood, 2023; Korzeniewski & Rossiter, 2020; McDougall et al., 2023; Poole et al., 1990). Together, these mechanisms are likely to contribute to the association between mitochondrial content and exercise tolerance (Jacobs et al., 2013, 2011).

## 3 PHYSIOLOGICAL STIMULI FOR ANGIOGENESIS AND MITOCHONDRIAL BIOGENESIS

Skeletal muscle and its vasculature exhibit remarkable plasticity in response to exposure to chronic exercise of sufficient duration and/or intensity. Since the early work of Andersen (1975) showing higher capillary density in trained versus untrained males, a plethora of research has demonstrated that aerobic training regimes elicit increases in capillary density that are evident in the early stages (≤4 weeks) of training (for meta‐analyses, see Liu et al., 2022; Mølmen et al., 2025; Rosenblat et al., 2022). Likewise, the seminal work of Holloszy (1967) demonstrating that progressive aerobic exercise training elicits substantial mitochondrial biogenesis has been followed by an abundance of research over subsequent decades with similar findings (for review, see Granata et al., 2018). Considering their beneficial impact on exercise tolerance highlighted above, there has been great interest in deducing the physiological and molecular stimuli that promote angiogenesis and mitochondrial biogenesis, with the ultimate aim of harnessing this knowledge to optimize exercise prescriptions and peripheral adaptations to aerobic exercise training. Although a comprehensive review of current understanding of these physiological stimuli is beyond the scope of this article, below is a brief account of the purported mechanisms by which chronic exercise exposure promotes angiogenesis and mitochondrial biogenesis. This section will provide essential context for the subsequent sections that examine the key physiological differences between large and small muscle mass exercise and their implications for promoting peripheral adaptations.

### 3.1 Angiogenesis

Angiogenesis, or capillary growth, occurs through two general mechanisms, namely sprouting and longitudinal splitting. Sprouting angiogenesis involves the growth of a new capillary out of an existing capillary, whereas splitting angiogenesis involves the intraluminal division of an existing capillary into two (Egginton, 2009; Hellsten & Gliemann, 2024). Both types of angiogenesis rely on the activation of pro‐angiogenic signalling compounds and pathways, with vascular endothelial growth factor recognized as being a crucial pro‐angiogenic factor owing to its potent mitogenic effect on endothelial cells (Petrova et al., 1999). However, the physiological and molecular stimuli for these two forms of angiogenesis appear to be distinct. Specifically, reductionist experimental approaches have revealed that sprouting is promoted through mechanical stretch and/or overload, which initiates a cascade of mechanotransduction events within muscle and endothelial cells, activation of proteolysis of the capillary basement membrane through metalloproteinases, abluminal sprout formation, proliferation and migration of endothelial cells and the formation of new microvessels (Egginton et al., 2001). In contrast, splitting is initiated through increases in capillary shear stress, i.e., the frictional force exerted on the capillary endothelium, which increases with blood flow velocity and elicits an increase in angiogenic factor release (Egginton et al., 2001). This response is thought to be regulated, at least in part, by the shear stress‐induced increase in nitric oxide synthase activity (Tuttle et al., 2001). Other stimuli, such as tissue hypoxia and the secretion of exercise‐associated metabolites such as lactate, are also thought to be important factors in promoting angiogenesis (Gorski & de Bock, 2019).

Aerobic‐type exercise induces all the abovementioned mechanisms that promote angiogenesis and thus represents the most potent stimulus for microvascular growth. However, the extent of these perturbations is dependent on the exercise intensity and duration. As such, it could be speculated that higher training intensities, which elicit higher blood flow, shear stress and metabolic perturbations, in addition to lower muscle and microvascular $P_{O_{2}}$, would provide a more potent stimulus for angiogenesis relative to lower training intensities. Indeed, a recent meta‐analysis found that continuous exercise performed at 51%–80% of ${\dot{V}}_{O_{2}\max}$ and high‐intensity interval training performed at 81%–100% of ${\dot{V}}_{O_{2}\max}$ elicited more pronounced increases in capillary density and capillary‐to‐fibre ratio relative to exercise performed at <50% of ${\dot{V}}_{O_{2}\max}$, with training intensity being of greater importance than training duration or frequency in the angiogenic response (Liu et al., 2022). However, it is important to note that several conflicting findings have been described throughout the literature in this regard, and there are contrasting suggestions that higher volume, lower intensity exercise might be equally effective or might even enhance capillary density better relative to brief high‐intensity efforts (Olfert et al., 2016). Although no consensus exists on the ‘optimal’ training stimulus for angiogenesis, and more research is required (Hellsten & Gliemann, 2024), it remains plausible that the greater increase in metabolic and mechanical stimuli associated with high‐intensity exercise could provide a more potent trigger for capillary growth.

### 3.2 Mitochondrial biogenesis

The molecular events leading to mitochondrial biogenesis in response to exercise have received considerable attention in recent years (for review, see Egan & Sharples, 2023). Briefly, although a myriad of exercise‐induced intracellular perturbations are likely to be implicated in initiating mitochondrial biogenesis, exercise‐induced changes in redox (NADH/NAD^+^) and phosphorylative potential (ATP/ADP × P_i_), increases in sarcoplasmic reticulum Ca^2+^ release and increases in reactive oxygen species are thought to be central in activating cellular transduction pathways (Coffey & Hawley, 2007). These stimuli activate AMP‐activated protein kinase, Ca^2+^/calmodulin‐dependent protein kinases and p38 mitogen‐activated protein kinase, which phosphorylate transcription factors and co‐activators, most notably peroxisome proliferator‐activated receptor‐γ coactivator‐1α (PGC‐1α) (McGee & Hargreaves, 2020). In turn, these events increase the potential for mRNA translation and the formation of mitochondrial proteins.

The optimal exercise stimulus in promoting mitochondrial biogenesis is uncertain and has been the subject of considerable debate (Bishop et al., 2019b; MacInnis et al., 2019). However, the magnitude of intracellular homeostatic perturbations, largely determined by exercise intensity, appears to play a central role. Indeed, studies have shown that the increases in the putative intracellular signalling responses that initiate mitochondrial biogenesis are associated with the degree of metabolic stress incurred during exercise (Egan et al., 2010; Fiorenza et al., 2018). For example, when comparing work‐matched low‐volume high‐intensity exercise, consisting of 18 × 5 s repeated sprints and 6 × 20 s maximal efforts, Fiorenza et al. (2018) found that the latter protocol was associated with greater metabolic perturbation (increases in muscle lactate and reductions in pH) and, concomitantly, greater increases in PGC‐1α mRNA relative to repeated sprints. Moreover, a recent meta‐analysis of training intervention studies found that increases in mitochondrial content per hour of exercise were positively associated with exercise intensity (Mølmen et al., 2025). The intensity dependence of mitochondrial biogenesis is consistent with the principle of hormesis, whereby disruptions in homeostasis elicit an adaptive response to minimize homeostatic perturbations in response to future stressors (Calabrese & Baldwin, 2002). In this context, higher‐intensity exercise induces greater metabolic disruption, which might act as a stronger signal to upregulate mitochondrial biogenesis and enhance cellular resilience.

## 4 ACUTE PERIPHERAL RESPONSES TO LARGE VERSUS SMALL MUSCLE MASS EXERCISE

**FIGURE 1:**
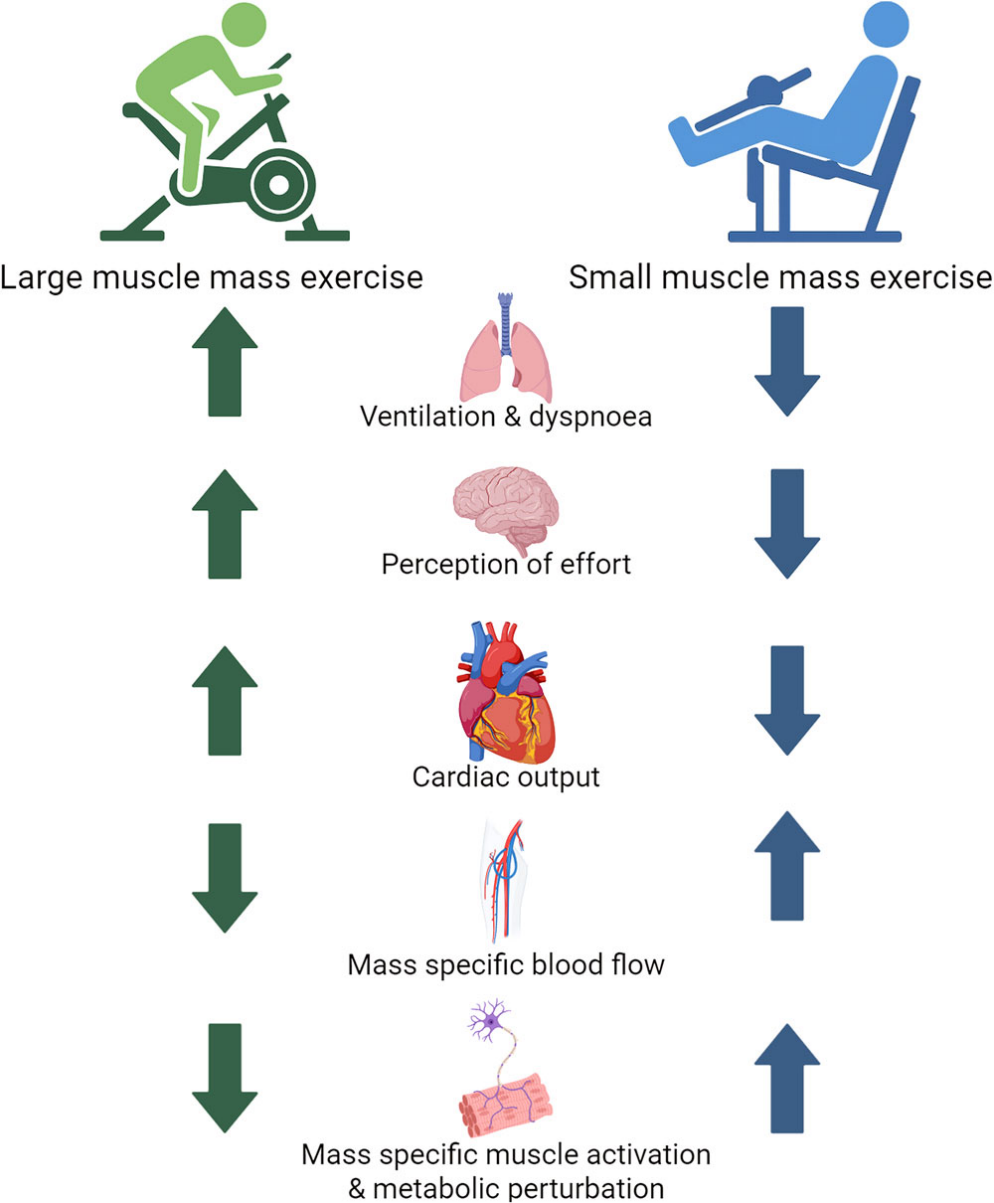
Overview of the key physiological differences between large and small muscle mass exercise. The reduction in active muscle mass during small muscle mass exercise reduces neural, pulmonary and cardiac constraints and is associated with a lower perception of effort and dyspnoea for a given mass‐specific metabolic rate. The attenuated central constraints permit a greater mass‐specific work rate, blood flow, muscle activation and metabolic disturbance, with potential implications for peripheral adaptations to repeated exercise exposure.

The conventional approach when prescribing exercise with the aim of inducing peripheral adaptations, such as angiogenesis and mitochondria biogenesis, is to use whole‐body, large muscle mass exercise, such as running or cycling. Although large muscle mass exercise is clearly effective in promoting peripheral adaptations, one limitation of this approach is that central constraints limit exercise tolerance and inhibit the increase in muscle blood flow and the metabolic stress incurred (Saltin, 1988). In this context, central constraints refer to physiological limitations arising upstream of the working muscles, primarily within the CNS, pulmonary and cardiac systems, that restrict the ability to deliver oxygen to locomotor muscles and sustain high‐intensity whole‐body exercise. In contrast, during small muscle mass exercise, such as single‐leg cycling or knee extension, these central constraints are substantially attenuated, permitting a greater mass‐specific work rate, muscle activation, blood flow and metabolic stress relative to large muscle mass exercise (Andersen & Saltin, 1985; Heidorn et al., 2023; Richardson & Saltin, 1998). In turn, this augmented local physiological and metabolic stress has the potential to promote the molecular signalling responses associated with angiogenesis and metabolic biogenesis beyond that associated with large muscle mass exercise (Abbiss et al., 2011). In this section, the evidence on differences in local blood flow and metabolic stress between small and large muscle mass exercise (summarized in Figure 1) and the physiological underpinnings of these differences are assessed.

### 4.1 Differences in mass‐specific blood flow between large and small muscle mass exercise

It is now well established that during small muscle mass exercise, peak mass‐specific muscle blood flow is substantially greater than that associated with large muscle mass exercise. This was first established by Andersen and Saltin (1985), who, using their single‐leg knee‐extension model, found that peak muscle blood flow was 250 mL (100 g)^−1^ min^−1^, values that were two to three times higher than had previously been reported in response to cycle ergometry (Richardson & Saltin, 1998; Richardson et al., 1993). As shown in Table 1, studies directly comparing muscle blood flow between small and large muscle mass exercise have unanimously shown substantially greater blood flow during the former modality. This higher mass‐specific blood flow permits greater mass‐specific muscle oxygen consumption, which Mortensen et al. (2005) reported as ∼45 and ∼10 mL (100 g)^−1^ min^−1^ during knee extension and cycling, respectively. Thus, substantial evidence indicates that peak mass‐specific blood flow and oxygen consumption are higher during small than large muscle mass exercise (Joyner, 2004).

**TABLE 1:** Peak blood flow during large muscle mass exercise, which consisted of double‐leg cycling in all studies, and small muscle mass exercise, which consisted of single‐leg dynamic knee extension (Cardinale et al., 2019; Mortensen et al., 2005) or single‐leg cycling (Klausen et al., 1982; LeJemtel et al., 1986).

Study	SMME modality	LMME modality	Blood flow during SMME	Blood flow during LMME
Mortensen et al. (2005)	Single‐leg knee extension	Double‐leg cycling	2.8 ± 0.3 L kg^−1^ min^−1^	0.9 ± 0.2 L kg^−1^ min^−1^
Cardinale et al. (2019)	Single‐leg knee extension	Double‐leg cycling	2.8 ± 6.3 L kg^−1^ min^−1^	1.2 ± 1.4 L kg^−1^ min^−1^
Klausen et al. (1982)	Single‐leg cycling	Double‐leg cycling	9.1 ± 1.8 L min^−1^	7.4 ± 0.3 L min^−1^
LeJemtel et al. (1986)	Single‐leg cycling	Double‐leg cycling	6.1 ± 0.8 L min^−1^	5.4 ± 0.8 L min^−1^

Note: Abbreviations: LMME, large muscle mass exercise; SMME, small muscle mass exercise.

The differences in mass‐specific blood flow between small and large muscle mass exercise can be explained, at least in part, by the lower cardiac constraints associated with small muscle mass exercise. Specifically, during large muscle mass exercise, the vasodilatory capacity of the vasculature supplying active muscles far exceeds the maximal pumping capacity of the heart (Volianitis & Secher, 2016). Consequently, although vascular conductance increases as exercise intensity goes from moderate to heavy, this rise must be curtailed as maximal cardiac output is approached in order to maintain arterial blood pressure (Mortensen et al., 2005). This regulation is achieved through ‘sympathetic restraint’, whereby noradrenaline release is accelerated at high exercise intensities (typically >∼80% ${\dot{V}}_{O_{2}\max}$). The exponential increase in noradrenaline release at high intensities shifts the balance between vasodilatory and vasoconstrictor influences away from the former, thereby preserving blood pressure when cardiac output nears its limit (Hughson et al., 1995). In contrast, during small muscle mass exercise, when cardiac output remains well within its limits, vascular conductance is ‘permitted’ to continue increasing with exercise intensity, with noradrenaline release being substantially lower than during large muscle mass exercise (Savard et al., 1989). Accordingly, the lack of sympathetic restraint required during small muscle mass exercise allows vascular conductance to rise in proportion to exercise intensity (Rowell, 1988).

A further potential contributor to higher vascular conductance and muscle blood flow during small muscle mass exercise is the lower ventilatory demands. It has been demonstrated that, during periods of high ventilatory work associated with high‐intensity large muscle mass exercise, inspiratory muscles preferentially ‘steal’ blood flow from working locomotor muscles through reflex vasoconstriction (Harms et al., 1997; Sheel et al., 2002). When inspiratory muscle work is decreased using assisted ventilation during high‐intensity cycling, there is a commensurate increase in vascular conductance and blood flow (Harms et al., 1997). However, during incremental single‐leg cycling performed to task failure, peak ventilation has been reported to be ∼80% of that associated with double‐leg incremental cycling (Zhang et al., 2021). Likewise, Rossman et al. (2012) showed that ventilation at task failure following single‐leg dynamic knee extension at 85% peak workload was ∼70% of that associated with cycling at the same relative intensity. It has been shown that these levels of ventilation are not associated with reflex vasoconstriction of locomotor muscles (Wetter et al., 1999). Consequently, reduced ventilatory work might contribute to higher blood flow during small muscle mass exercise.

### 4.2 Differences in muscle metabolic perturbation between large and small muscle mass exercise

**FIGURE 2:**
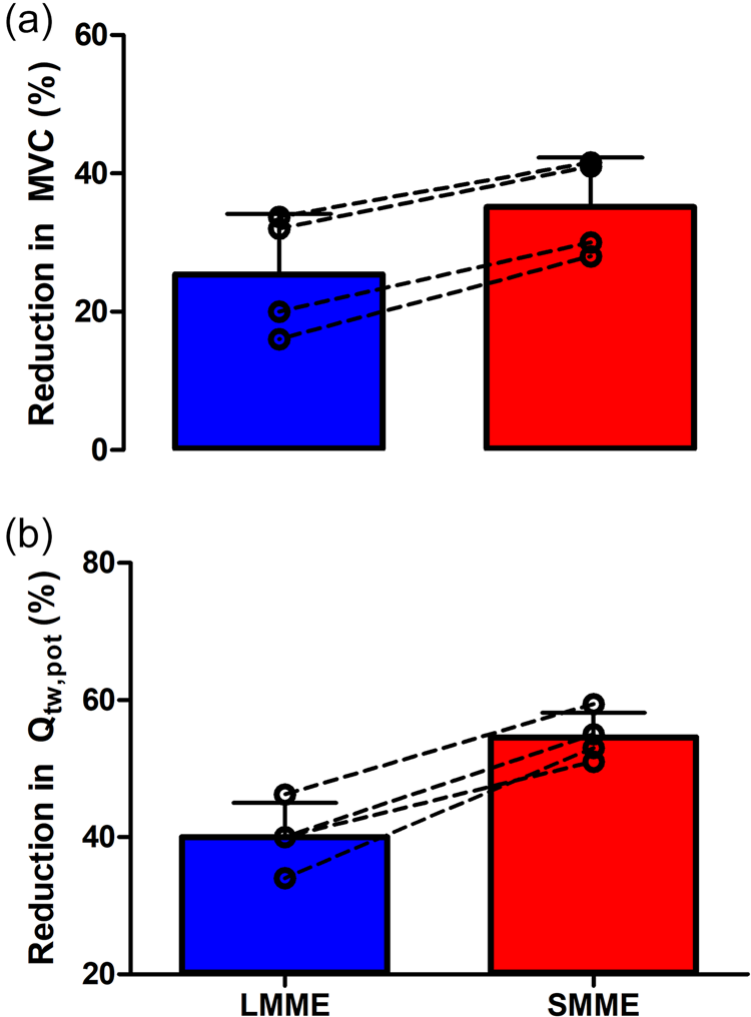
Reductions in maximal voluntary contraction (a) and resting potentiated twitch responses (b) derived from studies measuring the neuromuscular responses to high‐intensity large and small muscle mass exercise. All studies used cycling as the LMME modality, whereas SMME consisted of single‐leg cycling (Zhang et al., 2021, 2024) or single‐leg dynamic knee extension (Rossman et al., 2012; Weavil et al., 2018). Abbreviations: LMME, large muscle mass exercise; MVC, maximal voluntary contraction; *Q*_tw,pot_, potentiated twitch response; SMME, small muscle mass exercise.

In addition to the higher oxidative metabolism, mass‐specific substrate‐level phosphorylation also appears to be greater during small than large muscle mass exercise. Although few studies have compared muscle metabolic perturbations associated with substrate‐level phosphorylation between modalities (i.e., increases in muscle lactate, H^+^ and P_i_ and decreases in PCr), the net release of lactate during knee‐extensor exercise per unit of muscle is substantially higher during knee extension than cycling (Bangsbo et al., 1993; Richardson & Saltin, 1998). Moreover, several studies have shown that impairments in contractile function at task failure, measured through reductions in resting twitch responses to supramaximal nerve stimulation, are considerably higher during small than large muscle mass exercise (Figure 2; Rossman et al., 2012; Zhang et al., 2021, 2024; for a recent meta‐analysis, see Zhang et al., 2025). Given that impairments in contractile function are induced by the build‐up of metabolites associated with substrate‐level phosphorylation, such as P_i_ and H^+^ (Allen & Trajanovska, 2012; Hureau et al., 2022; Sundberg & Fitts, 2019), these results indicate that the magnitude of muscle metabolic perturbation is greater during small than large muscle mass exercise.

Different theories have been posited to explain the greater metabolic perturbation and contractile impairment at task failure during small muscle mass exercise. For example, Rossman et al. (2012) suggested that during small muscle mass exercise, the lower ‘ensemble’ group III/IV afferent feedback, which is thought to inhibit central drive and voluntary activation during exercise (Sidhu et al., 2017), permits greater muscle activation and thereby accentuates local metabolic demands. Indeed, research has consistently shown higher EMG at task failure during small than large muscle mass exercise (Rossman et al., 2012; Zhang et al., 2021). However, studies have generally shown negligible differences in voluntary activation between modalities when measured using the interpolation twitch method (Zhang et al., 2025), which does not align with the suggestion of greater inhibition of central drive during large muscle mass exercise. More recently, Thomas et al. (2018) suggested that the attenuated overall challenge to homeostasis during small muscle mass exercise, occurring as a result of the reduced cardiorespiratory demands, reduces sensory input and permits a greater degree of local metabolic stress to be tolerated before the perception of effort becomes excessive. Such a scenario is plausible given the lower corollary discharge and reduced exertional dyspnoea associated with small muscle mass exercise, both of which are important contributors to the perception of effort (Amann et al., 2007; de Morree et al., 2012). Indeed, previous work has shown that the perception of effort is lower during single‐ than double‐leg cycling at similar mass‐specific power outputs (Abbiss et al., 2011; MacInnis et al., 2017). Thus, the greater metabolic perturbation observed during small muscle mass exercise is likely to reflect the combination of reduced cardiorespiratory strain and sensory input, which permits greater muscle activation and tolerance of local metabolic stress before task failure.

## 5 EVIDENCE ON THE EFFICACY OF SMALL MUSCLE MASS EXERCISE IN PROMOTING PERIPHERAL ADAPTATIONS

As highlighted above, both muscle blood flow (and the associated shear stress) and the degree of metabolic perturbation (key stimuli for angiogenesis and mitochondrial biogenesis, respectively) are markedly greater during small than large muscle mass exercise. Given that the magnitude of these perturbations is likely to influence subsequent transcriptional responses (Fiorenza et al., 2020, 2018), it is conceivable that intense small muscle mass aerobic exercise could prove a particularly potent stimulus for capillary growth and mitochondrial biogenesis. Unfortunately, however, research comparing peripheral adaptations between modalities is scarce. In one study in trained cyclists, Abbiss et al. (2011) had participants perform 3 weeks of high‐intensity single‐ and double‐leg cycling consisting of 3 × 4 min maximal self‐paced intervals. Following the interventions, the improvements in markers of mitochondrial oxidative capacity (cytochrome *c* oxidase subunits II and IV) were greater for single‐ than double‐leg cycling. These findings were attributed to the greater mass‐specific work achieved during single‐ than double‐leg cycling, which is consistent with previous research using self‐paced interval exercise with these two modalities (Haddad et al., 2024). Although Mølmen et al. (2019) found no additional improvement in oxidative enzymes or capillary density following small (single‐leg knee extension) and large muscle mass exercise (single‐leg knee extension with arm cycling), the work rate and duration of exercise imposed during knee extension were matched between conditions, which probably negated the elevated metabolic stress and mass‐specific blood flow usually observed with small muscle mass exercise. In patients with heart failure with reduced ejection fraction, Tyni‐Lenné et al. (1999) found that 8 weeks of continuous knee extension and cycling training performed at 50%–70% of peak work rate produced increases in citrate synthase of 45% and 23%, respectively, although the difference did not reach statistical significance. Thus, although preliminary evidence suggests that small muscle mass aerobic exercise might elicit greater improvements in mitochondrial function than large muscle mass exercise, further research is needed to confirm these findings.

In addition, several studies have examined small muscle mass exercise alone and reported substantial increases in mitochondrial content and capillary density. Following 6 weeks of single‐leg knee‐extension training in healthy individuals, Skattebo et al. (2020b) found that citrate synthase and the capillary‐to‐fibre ratio were 45% and 18% higher, respectively, in the trained compared with untrained leg, with other studies producing similar findings (Blomstrand et al., 2011; Kiens et al., 1993; Rud et al., 2012). In patients with heart failure with reduced ejection fraction, Magnusson et al. (1996) reported increases in capillary‐to‐fibre ratio of 47% in response to 8 weeks of knee‐extensor exercise, whilst citrate synthase increased by 77%. Likewise, Esposito et al. (2011) found that the capillary‐to‐fibre ratio increased from 1.6 to 1.8 and mitochondrial volume density increased from 3.8% to 4.9% in response to 8 weeks of knee‐extensor exercise in individuals with heart failure with reduced ejection fraction. Accordingly, evidence clearly supports the efficacy of small muscle mass exercise in promoting angiogenesis and increases in mitochondrial content.

The efficacy of small muscle mass exercise hinges on its ability to improve performance during conventional whole‐body exercise. However, translating the local adaptations induced by small muscle mass exercise of locomotor muscles into improvements in whole‐body performance would probably require training both limbs separately, although some research has shown improvements in cycle performance after training only one leg (Esposito et al., 2011; Skattebo et al., 2020b). Few studies have taken this approach. In healthy middle‐aged adults, Gordon et al. (2019) reported that 8 weeks of high‐intensity single‐leg cycling (both legs trained separately) increased peak power output during double‐leg cycling by 22%, which was similar to improvements following both moderate‐ and high‐intensity double‐leg cycling. In well‐trained cyclists who performed single‐leg cycling training with both legs, Abbiss et al. (2011) found no improvement in peak power output or time‐trial performance. However, the intervention was limited to only six sessions over 3 weeks, and double‐leg cycling also failed to improve these variables in the same study. In patients with chronic obstructive pulmonary disease, single‐leg cycling training of both legs individually over 7 weeks resulted in a 35% improvement in peak power output during conventional cycling (Dolmage & Goldstein, 2008), which was greater than the 11% increase following double‐leg cycling training, with Bjørgen et al. (2009) producing similar results. Likewise, in two studies conducted in patients with chronic heart failure, greater improvements in cycling peak power output were observed following 8 weeks of knee‐extension training than traditional cycling training (Gordon et al., 1999; Tyni‐Lenné et al., 1999). Thus, although evidence in healthy individuals is limited, studies in patients with respiratory and cardiac disorders consistently show that small muscle mass exercise can enhance whole‐body performance, possibly more so than whole‐body exercise.

## 6 FUTURE RESEARCH AND POTENTIAL APPLICATIONS OF SMALL MUSCLE MASS AEROBIC EXERCISE

Although preliminary research indicates that peripheral adaptations might be greater enhanced following small than large muscle mass aerobic exercise (Abbiss et al., 2011; Tyni‐Lenné et al., 1999), direct comparisons between modalities remain scarce. Further research is required to examine both the acute signalling pathways activated by small versus large muscle mass exercise and the chronic vascular and metabolic adaptations that result from repeated exposure. In addition, the translation of these adaptations to improvements in exercise performance and its submaximal determinants (e.g., lactate threshold and critical power) following small muscle mass requires further investigation. Such research is imperative to understand whether the peripheral adaptations induced by small muscle mass exercise translate into meaningful improvements in whole‐body endurance performance, rather than representing isolated local changes without functional benefit (Esposito et al., 2011).

Although much has been learned about the differences in the acute physiological responses to small and large muscle mass exercise, the focus of this research has predominantly been on peak exercise responses, such as peak mass‐specific work rate, blood flow and ${\dot{V}}_{O_{2}}$ (Cardinale et al., 2019; Mortensen et al., 2005; Richardson et al., 1993). In contrast, less research has assessed whether differences in the mass‐specific work rate, ${\dot{V}}_{O_{2}}$ and/or muscle activation associated with submaximal determinants of exercise tolerance (e.g., the lactate threshold, critical power and e.g., the lactate threshold and critical power) exist between modalities, although recent research found higher mass‐specific maximal fat oxidation during single‐ than double‐leg cycling (Skattebo et al., 2022). Such research is warranted to provide further insight into the mechanistic bases of small muscle mass exercise in improving peripheral vascular and metabolic function.

The potential applications of small muscle mass exercise are unlikely to be confined to a single population. However, this training modality has shown particular promise in those with cardiac and respiratory limitations, whose central constraints severely limit exercise tolerance during whole‐body exercise (Bjørgen et al., 2009; Dolmage & Goldstein, 2008; Gordon et al., 1999; Tyni‐Lenné et al., 1999). Further research is also warranted in highly trained endurance athletes, who display attenuated adaptive responses to conventional aerobic training owing to their already high training loads (Granata et al., 2020). In this context, small muscle mass exercise, through the augmented metabolic perturbation it elicits, might represent a useful training variation capable of providing an additional adaptive stimulus. Future research on the efficacy of small muscle mass exercise might also consider focusing on elderly individuals, who exhibit reduced cardiac performance and increased work of breathing (Ferrari et al., 2003; Weavil et al., 2022), which is likely to contribute to exercise intolerance during whole‐body exercise. These areas, amongst others, provide fruitful avenues for further research

Finally, it is important to note that, although small muscle mass exercise could provide a potent stimulus for peripheral adaptation, the low demands for cardiac output mean that central haemodynamic adaptations to this training modality might be limited. Previous work using small muscle mass training in patient populations has observed no improvement in maximal cardiac output (del Torto et al., 2021; Esposito et al., 2011; Hearon Jr et al., 2022). Thus, when central haemodynamic adaptations are sought, training modalities that elicit substantially higher cardiac output, such as large muscle mass endurance exercise or interval training involving whole‐body exercise, are likely to be necessary to provide an adequate stimulus for central cardiovascular remodelling. Accordingly, an interesting area for future research is to assess how small and large muscle mass exercise training could be combined in order to optimize adaptations at both the central and peripheral levels.

## 7 CONCLUSION

Aerobic exercise performed with a small muscle mass, such as single‐leg cycling or knee extension, reduces the cardiac, ventilatory and neural constraints typically associated with large muscle mass exercise. The attenuation of these central demands permits greater mass‐specific muscle activation, blood flow, metabolic perturbation and exercise intensity to be achieved. These stressors are considered key stimuli initiating transcriptional responses that underpin improvements in vascular and metabolic function. Consequently, small muscle mass aerobic exercise might represent a viable strategy to elicit peripheral adaptations, such as angiogenesis and mitochondrial biogenesis, that contribute to enhanced exercise tolerance. Studies in clinical populations with ventilatory or cardiac limitations have reported encouraging results regarding the efficacy and feasibility of small muscle mass exercise training. However, despite these apparent physiological advantages, the relative efficacy of small and large muscle mass exercise in driving peripheral adaptations remains largely unexplored. Clarifying these differences will help to determine the extent to which small muscle mass exercise can serve as an effective model or training modality for enhancing peripheral function in performance, health and disease.

## AUTHOR CONTRIBUTIONS

Sole author.

## CONFLICT OF INTEREST

None declared.

## FUNDING INFORMATION

None.

## References

[eph70183-bib-0001] Abbiss, C. R. , Karagounis, L. G. , Laursen, P. B. , Peiffer, J. J. , Martin, D. T. , Hawley, J. A. , Fatehee, N. N. , & Martin, J. C. (2011). Single‐leg cycle training is superior to double‐leg cycling in improving the oxidative potential and metabolic profile of trained skeletal muscle. Journal of Applied Physiology, 110(5), 1248–1255. 21330612 10.1152/japplphysiol.01247.2010

[eph70183-bib-0002] Allen, D. G. , & Trajanovska, S. (2012). The multiple roles of phosphate in muscle fatigue. Frontiers in Physiology, 3, 463. 23248600 10.3389/fphys.2012.00463 PMC3518787

[eph70183-bib-0003] Amann, M. , Pegelow, D. F. , Jacques, A. J. , & Dempsey, J. A. (2007). Inspiratory muscle work in acute hypoxia influences locomotor muscle fatigue and exercise performance of healthy humans. American Journal of Physiology‐Regulatory, Integrative and Comparative Physiology, 293(5), R2036–R2045. 17715180 10.1152/ajpregu.00442.2007

[eph70183-bib-0004] Andersen, P. (1975). Capillary density in skeletal muscle of man. Acta Physiologica Scandinavica, 95(2), 203–205. 127508 10.1111/j.1748-1716.1975.tb10043.x

[eph70183-bib-0005] Andersen, P. , & Saltin, B. (1985). Maximal perfusion of skeletal muscle in man. The Journal of Physiology, 366(1), 233–249. 4057091 10.1113/jphysiol.1985.sp015794 PMC1193029

[eph70183-bib-0006] Bachasson, D. , Decorte, N. , Wuyam, B. , Millet, G. Y. , & Verges, S. (2016). Original Research: Central and peripheral quadriceps fatigue in young and middle‐aged untrained and endurance‐trained men: A comparative study. Experimental Biology and Medicine, 241(16), 1844–1852. 27287015 10.1177/1535370216654225 PMC5027946

[eph70183-bib-0007] Balaban, R. S. (1990). Regulation of oxidative phosphorylation in the mammalian cell. American Journal of Physiology, 258(3), C377–C389. 2138418 10.1152/ajpcell.1990.258.3.C377

[eph70183-bib-0008] Bangsbo, J. , Johansen, L. , Graham, T. , & Saltin, B. (1993). Lactate and H+ effluxes from human skeletal muscles during intense, dynamic exercise. The Journal of Physiology, 462(1), 115–133. 8331579 10.1113/jphysiol.1993.sp019546 PMC1175292

[eph70183-bib-0009] Bergström, J. , Hermansen, L. , Hultman, E. , & Saltin, B. (1967). Diet, muscle glycogen and physical performance. Acta Physiologica Scandinavica, 71(2–3), 140–150. 5584523 10.1111/j.1748-1716.1967.tb03720.x

[eph70183-bib-0010] Bishop, D. J. , Botella, J. , Genders, A. J. , Lee, M. J.‐C. , Saner, N. J. , Kuang, J. , Yan, X. , & Granata, C. (2019a). High‐intensity exercise and mitochondrial biogenesis: Current controversies and future research directions. Physiology, 34(1), 56–70. 30540234 10.1152/physiol.00038.2018

[eph70183-bib-0011] Bishop, D. J. , Botella, J. , & Granata, C. (2019b). CrossTalk opposing view: Exercise training volume is more important than training intensity to promote increases in mitochondrial content. The Journal of Physiology, 597(16), 4115–4118. 31309570 10.1113/JP277634

[eph70183-bib-0012] Bjørgen, S. , Hoff, J. , Husby, V. S. , Høydal, M. A. , Tjønna, A. E. , Steinshamn, S. , Richardson, R. S. , & Helgerud, J. (2009). Aerobic high intensity one and two legs interval cycling in chronic obstructive pulmonary disease: The sum of the parts is greater than the whole. European Journal of Applied Physiology, 106, 501–507. 19337746 10.1007/s00421-009-1038-1

[eph70183-bib-0013] Blomstrand, E. , Krustrup, P. , Søndergaard, H. , Rådegran, G. , Calbet, J. A. , & Saltin, B. (2011). Exercise training induces similar elevations in the activity of oxoglutarate dehydrogenase and peak oxygen uptake in the human quadriceps muscle. Pflugers Archiv: European Journal of Physiology, 462(2), 257–265. 21611730 10.1007/s00424-011-0978-6

[eph70183-bib-0014] Boushel, R. , Gnaiger, E. , Calbet, J. A. , Gonzalez‐Alonso, J. , Wright‐Paradis, C. , Sondergaard, H. , Ara, I. , Helge, J. W. , & Saltin, B. (2011). Muscle mitochondrial capacity exceeds maximal oxygen delivery in humans. Mitochondrion, 11(2), 303–307. 21147270 10.1016/j.mito.2010.12.006

[eph70183-bib-0015] Broxterman, R. M. , Wagner, P. D. , & Richardson, R. S. (2021). Exercise training in COPD: Muscle O(2) transport plasticity. European Respiratory Journal, 58(2), 2004146. 33446612 10.1183/13993003.04146-2020

[eph70183-bib-0016] Calabrese, E. J. , & Baldwin, L. A. (2002). Defining hormesis. Human, & Experimental Toxicology, 21(2), 91–97. 12102503 10.1191/0960327102ht217oa

[eph70183-bib-0017] Cardinale, D. A. , Larsen, F. J. , Jensen‐Urstad, M. , Rullman, E. , Søndergaard, H. , Morales‐Alamo, D. , Ekblom, B. , Calbet, J. A. L. , & Boushel, R. (2019). Muscle mass and inspired oxygen influence oxygen extraction at maximal exercise: Role of mitochondrial oxygen affinity. Acta Physiologica, 225(1), e13110. 29863764 10.1111/apha.13110

[eph70183-bib-0018] Chance, B. , & Williams, G. R. (1955). Respiratory enzymes in oxidative phosphorylation. I. Kinetics of oxygen utilization. Journal of Biological Chemistry, 217(1), 383–393. 13271402

[eph70183-bib-0019] Clanton, T. L. , Hogan, M. C. , & Gladden, L. B. (2013). Regulation of cellular gas exchange, oxygen sensing, and metabolic control. Comprehensive Physiology, 3(3), 1135–1190. 23897683 10.1002/cphy.c120030

[eph70183-bib-0020] Coffey, V. G. , & Hawley, J. A. (2007). The molecular bases of training adaptation. Sports Medicine, 37(9), 737–763. 17722947 10.2165/00007256-200737090-00001

[eph70183-bib-0021] Coyle, E. F. , Coggan, A. R. , Hopper, M. K. , & Walters, T. J. (1988). Determinants of endurance in well‐trained cyclists. Journal of Applied Physiology, 64(6), 2622–2630. 3403447 10.1152/jappl.1988.64.6.2622

[eph70183-bib-0022] Davies, C. T. , & Sargeant, A. J. (1975). Effects of training on the physiological responses to one‐ and two‐leg work. Journal of Applied Physiology, 38(3), 377–375. 1150549 10.1152/jappl.1975.38.3.377

[eph70183-bib-0023] del Torto, A. , Capelli, C. , Peressutti, R. , di Silvestre, A. , Livi, U. , Nalli, C. , Sponga, S. , Amici, G. , Baccarani, U. , & Lazzer, S. (2021). Effect of small vs large muscle mass endurance training on maximal oxygen uptake in organ transplanted recipients. Applied Physiology, Nutrition and Metabolism, 46, 994–1003. 10.1139/apnm-2020-0987 34315281

[eph70183-bib-0024] de Morree, H. M. , Klein, C. , & Marcora, S. M. (2012). Perception of effort reflects central motor command during movement execution. Psychophysiology, 49(9), 1242–1253. 22725828 10.1111/j.1469-8986.2012.01399.x

[eph70183-bib-0025] Dolmage, T. E. , & Goldstein, R. S. (2008). Effects of one‐legged exercise training of patients with COPD. Chest, 133(2), 370–376. 17925417 10.1378/chest.07-1423

[eph70183-bib-0026] Ducrocq, G. P. , Hureau, T. J. , Bøgseth, T. , Meste, O. , & Blain, G. M. (2021). Recovery from fatigue after cycling time trials in elite endurance athletes. Medicine and Science in Sports and Exercise, 53(5), 904–917. 33148973 10.1249/MSS.0000000000002557

[eph70183-bib-0027] Dudley, G. A. , Tullson, P. C. , & Terjung, R. L. (1987). Influence of mitochondrial content on the sensitivity of respiratory control. Journal of Biological Chemistry, 262(19), 9109–9114. 3597408

[eph70183-bib-0028] Egan, B. , Carson, B. P. , Garcia‐Roves, P. M. , Chibalin, A. V. , Sarsfield, F. M. , Barron, N. , McCaffrey, N. , Moyna, N. M. , Zierath, J. R. , & O'Gorman, D. J. (2010). Exercise intensity‐dependent regulation of peroxisome proliferator‐activated receptor coactivator‐1 mRNA abundance is associated with differential activation of upstream signalling kinases in human skeletal muscle. The Journal of Physiology, 588(10), 1779–1790. 20308248 10.1113/jphysiol.2010.188011 PMC2887994

[eph70183-bib-0029] Egan, B. , & Sharples, A. P. (2023). Molecular responses to acute exercise and their relevance for adaptations in skeletal muscle to exercise training. Physiological Reviews, 103(3), 2057–2170. 36395350 10.1152/physrev.00054.2021

[eph70183-bib-0030] Egginton, S. (2009). Invited review: Activity‐induced angiogenesis. Pflugers Archiv: European Journal of Physiology, 457(5), 963–977. 18704490 10.1007/s00424-008-0563-9

[eph70183-bib-0031] Egginton, S. , Zhou, A. L. , Brown, M. D. , & Hudlická, O. (2001). Unorthodox angiogenesis in skeletal muscle. Cardiovascular Research, 49(3), 634–646. 11166277 10.1016/s0008-6363(00)00282-0

[eph70183-bib-0032] Esposito, F. , Mathieu‐Costello, O. , Wagner, P. D. , & Richardson, R. S. (2018). Acute and chronic exercise in patients with heart failure with reduced ejection fraction: Evidence of structural and functional plasticity and intact angiogenic signalling in skeletal muscle. The Journal of Physiology, 596(21), 5149–5161. 30192995 10.1113/JP276678 PMC6209757

[eph70183-bib-0033] Esposito, F. , Reese, V. , Shabetai, R. , Wagner, P. D. , & Richardson, R. S. (2011). Isolated quadriceps training increases maximal exercise capacity in chronic heart failure: The role of skeletal muscle convective and diffusive oxygen transport. Journal of the American College of Cardiology, 58(13), 1353–1362. 21920265 10.1016/j.jacc.2011.06.025 PMC3180857

[eph70183-bib-0034] Ferrari, A. U. , Radaelli, A. , & Centola, M. (2003). Invited review: Aging and the cardiovascular system. Journal of Applied Physiology (1985), 95(6), 2591–2597. 10.1152/japplphysiol.00601.2003 14600164

[eph70183-bib-0035] Fiorenza, M. , Gliemann, L. , Brandt, N. , & Bangsbo, J. (2020). Hormetic modulation of angiogenic factors by exercise‐induced mechanical and metabolic stress in human skeletal muscle. American Journal of Physiology‐Heart and Circulatory Physiology, 319(4), H824–H834. 32822216 10.1152/ajpheart.00432.2020

[eph70183-bib-0036] Fiorenza, M. , Gunnarsson, T. P. , Hostrup, M. , Iaia, F. M. , Schena, F. , Pilegaard, H. , & Bangsbo, J. (2018). Metabolic stress‐dependent regulation of the mitochondrial biogenic molecular response to high‐intensity exercise in human skeletal muscle. The Journal of Physiology, 596(14), 2823–2840. 29727016 10.1113/JP275972 PMC6046074

[eph70183-bib-0037] Gifford, J. R. , Garten, R. S. , Nelson, A. D. , Trinity, J. D. , Layec, G. , Witman, M. A. , Weavil, J. C. , Mangum, T. , Hart, C. , Etheredge, C. , Jessop, J. , Bledsoe, A. , Morgan, D. E. , Wray, D. W. , Rossman, M. J. , & Richardson, R. S. (2016). Symmorphosis and skeletal muscle ${\dot{V}}_{O_{2}\max}$: *In vivo* and *in vitro* measures reveal differing constraints in the exercise‐trained and untrained human. The Journal of Physiology, 594(6), 1741–1751. 26614395 10.1113/JP271229 PMC4799962

[eph70183-bib-0038] Gollnick, P. D. , & Saltin, B. (1982). Significance of skeletal muscle oxidative enzyme enhancement with endurance training. Clinical Physiology, 2(1), 1–12. 7201906 10.1111/j.1475-097x.1982.tb00001.x

[eph70183-bib-0039] Gordon, A. , Tyni‐Lenné, R. , Jansson, E. , Jensen‐Urstad, M. , & Kaijser, L. (1999). Beneficial effects of exercise training in heart failure patients with low cardiac output response to exercise—a comparison of two training models. Journal of Internal Medicine, 246(2), 175–182. 10447786 10.1046/j.1365-2796.1999.00555.x

[eph70183-bib-0040] Gordon, N. , Abbiss, C. R. , Maiorana, A. J. , James, A. P. , Clark, K. , Marston, K. J. , & Peiffer, J. J. (2019). High‐intensity single‐leg cycling improves cardiovascular disease risk factor profile. Medicine and Science in Sports and Exercise, 51(11), 2234–2242. 31145180 10.1249/MSS.0000000000002053

[eph70183-bib-0041] Gorski, T. , & de Bock, K. (2019). Metabolic regulation of exercise‐induced angiogenesis. Vascular Biology, 1(1), H1–H8. 32923947 10.1530/VB-19-0008 PMC7439921

[eph70183-bib-0042] Goulding, R. P. , & Marwood, S. (2023). Interaction of factors determining critical power. Sports Medicine, 53(3), 595–613. 36622556 10.1007/s40279-022-01805-w PMC9935749

[eph70183-bib-0043] Granata, C. , Jamnick, N. A. , & Bishop, D. J. (2018). Training‐induced changes in mitochondrial content and respiratory function in human skeletal muscle. Sports Medicine, 48(8), 1809–1828. 29934848 10.1007/s40279-018-0936-y

[eph70183-bib-0044] Granata, C. , Oliveira, R. S. F. , Little, J. P. , & Bishop, D. J. (2020). Forty high‐intensity interval training sessions blunt exercise‐induced changes in the nuclear protein content of PGC‐1α and p53 in human skeletal muscle. American Journal of Physiology‐Endocrinology and Metabolism, 318(2), E224–E236. 31794264 10.1152/ajpendo.00233.2019 PMC7052577

[eph70183-bib-0045] Green, D. J. , Hopman, M. T. , Padilla, J. , Laughlin, M. H. , & Thijssen, D. H. (2017). Vascular adaptation to exercise in humans: Role of hemodynamic stimuli. Physiological Reviews, 97(2), 495–528. 28151424 10.1152/physrev.00014.2016 PMC5539408

[eph70183-bib-0046] Green, H. J. , Burnett, M. , Kollias, H. , Ouyang, J. , Smith, I. , & Tupling, S. (2012). Can increases in capillarization explain the early adaptations in metabolic regulation in human muscle to short‐term training? Canadian Journal of Physiology and Pharmacology, 90(5), 557–566. 22471991 10.1139/y2012-013

[eph70183-bib-0047] Green, H. J. , Cadefau, J. , Cussó, R. , Ball‐Burnett, M. , & Jamieson, G. (1995a). Metabolic adaptations to short‐term training are expressed early in submaximal exercise. Canadian Journal of Physiology and Pharmacology, 73(4), 474–482. 7671190 10.1139/y95-060

[eph70183-bib-0048] Green, H. J. , Jones, S. , Ball‐Burnett, M. , Farrance, B. , & Ranney, D. (1995b). Adaptations in muscle metabolism to prolonged voluntary exercise and training. Journal of Applied Physiology, 78(1), 138–145. 7713803 10.1152/jappl.1995.78.1.138

[eph70183-bib-0049] Haddad, T. , Spence, A. L. , Peiffer, J. , Blain, G. M. , Brisswalter, J. , & Abbiss, C. R. (2024). The improvement in exercise performance during reduced muscle mass exercise is associated with an increase in femoral blood flow in older and younger endurance‐trained athletes. Journal of Sports Science and Medicine, 23, 46–55. 38455445 10.52082/jssm.2024.46 PMC10915611

[eph70183-bib-0050] Harms, C. A. , Babcock, M. A. , McClaran, S. R. , Pegelow, D. F. , Nickele, G. A. , Nelson, W. B. , & Dempsey, J. A. (1997). Respiratory muscle work compromises leg blood flow during maximal exercise. Journal of Applied Physiology, 82(5), 1573–1583. 9134907 10.1152/jappl.1997.82.5.1573

[eph70183-bib-0051] Hearon, Jr. C. M. , Samels, M. , Dias, K. A. , MacNamara, J. P. , Levine, B. D. , & Sarma, S. (2022). Isolated knee extensor exercise training improves skeletal muscle vasodilation, blood flow, and functional capacity in patients with HFpEF. Physiological Reports, 10(15), e15419. 35924338 10.14814/phy2.15419 PMC9350466

[eph70183-bib-0052] Heidorn, C. E. , Elmer, S. J. , Wehmanen, K. W. , Martin, J. C. , & McDaniel, J. (2023). Single‐leg cycling to maintain and improve function in healthy and clinical populations. Frontiers in Physiology, 14, 1105772. 37187959 10.3389/fphys.2023.1105772 PMC10175616

[eph70183-bib-0053] Hellsten, Y. , & Gliemann, L. (2024). Peripheral limitations for performance: Muscle capillarization. Scandinavian Journal of Medicine, & Science in Sports, 34(1), e14442. 37770233 10.1111/sms.14442

[eph70183-bib-0054] Hellsten, Y. , & Nyberg, M. (2015). Cardiovascular adaptations to exercise training. Comprehensive Physiology, 6(1), 1–32. 26756625 10.1002/cphy.c140080

[eph70183-bib-0055] Hogan, M. C. , Arthur, P. G. , Bebout, D. E. , Hochachka, P. W. , & Wagner, P. D. (1992). Role of O2 in regulating tissue respiration in dog muscle working in situ. Journal of Applied Physiology (1985), 73(2), 728–736. 10.1152/jappl.1992.73.2.728 1400003

[eph70183-bib-0056] Hogan, M. C. , Gladden, L. B. , Grassi, B. , Stary, C. M. , & Samaja, M. (1998). Bioenergetics of contracting skeletal muscle after partial reduction of blood flow. Journal of Applied Physiology (1985), 84(6), 1882–1888. 10.1152/jappl.1998.84.6.1882 9609780

[eph70183-bib-0057] Holloszy, J. O. (1967). Biochemical adaptations in muscle. Effects of exercise on mitochondrial oxygen uptake and respiratory enzyme activity in skeletal muscle. Journal of Biological Chemistry, 242(9), 2278–2282. 4290225

[eph70183-bib-0058] Hoppeler, H. , Howald, H. , Conley, K. , Lindstedt, S. L. , Claassen, H. , Vock, P. , & Weibel, E. R. (1985). Endurance training in humans: Aerobic capacity and structure of skeletal muscle. Journal of Applied Physiology, 59(2), 320–327. 4030584 10.1152/jappl.1985.59.2.320

[eph70183-bib-0059] Hughson, R. L. , Green, H. J. , & Sharratt, M. T. (1995). Gas exchange, blood lactate, and plasma catecholamines during incremental exercise in hypoxia and normoxia. Journal of Applied Physiology, 79(4), 1134–1141. 8567554 10.1152/jappl.1995.79.4.1134

[eph70183-bib-0060] Hughson, R. L. , Tschakovsky, M. E. , & Houston, M. E. (2001). Regulation of oxygen consumption at the onset of exercise. Exercise and Sport Sciences Reviews, 29(3), 129–133. 11474961 10.1097/00003677-200107000-00008

[eph70183-bib-0061] Hureau, T. J. , Broxterman, R. M. , Weavil, J. C. , Lewis, M. T. , Layec, G. , & Amann, M. (2022). On the role of skeletal muscle acidosis and inorganic phosphates as determinants of central and peripheral fatigue: A (31) P‐MRS study. The Journal of Physiology, 600(13), 3069–3081. 35593645 10.1113/JP283036 PMC9250628

[eph70183-bib-0062] Iaia, F. M. , Perez‐Gomez, J. , Thomassen, M. , Nordsborg, N. B. , Hellsten, Y. , & Bangsbo, J. (2011). Relationship between performance at different exercise intensities and skeletal muscle characteristics. Journal of Applied Physiology, 110(6), 1555–1563. 21436467 10.1152/japplphysiol.00420.2010

[eph70183-bib-0063] Ingjer, F. (1979). Capillary supply and mitochondrial content of different skeletal muscle fiber types in untrained and endurance‐trained men. A histochemical and ultrastructural study. European Journal of Applied Physiology and Occupational Physiology, 40(3), 197–209. 421683 10.1007/BF00426942

[eph70183-bib-0064] Jacobs, R. A. , Flück, D. , Bonne, T. C. , Bürgi, S. , Christensen, P. M. , Toigo, M. , & Lundby, C. (2013). Improvements in exercise performance with high‐intensity interval training coincide with an increase in skeletal muscle mitochondrial content and function. Journal of Applied Physiology, 115(6), 785–793. 23788574 10.1152/japplphysiol.00445.2013

[eph70183-bib-0065] Jacobs, R. A. , Rasmussen, P. , Siebenmann, C. , Díaz, V. , Gassmann, M. , Pesta, D. , Gnaiger, E. , Nordsborg, N. B. , Robach, P. , & Lundby, C. (2011). Determinants of time trial performance and maximal incremental exercise in highly trained endurance athletes. Journal of Applied Physiology, 111(5), 1422–1430. 21885805 10.1152/japplphysiol.00625.2011

[eph70183-bib-0066] Joyner, M. J. (2004). Feeding the sleeping giant: Muscle blood flow during whole body exercise. The Journal of Physiology, 558(1), 1. 15155795 10.1113/jphysiol.2004.067306 PMC1664931

[eph70183-bib-0067] Katz, A. , & Sahlin, K. (1987). Effect of decreased oxygen availability on NADH and lactate contents in human skeletal muscle during exercise. Acta Physiologica Scandinavica, 131(1), 119–127. 3673605 10.1111/j.1748-1716.1987.tb08213.x

[eph70183-bib-0068] Katz, A. , & Sahlin, K. (1990). Role of oxygen in regulation of glycolysis and lactate production in human skeletal muscle. Exercise and Sport Sciences Reviews, 18(1), 1–28. 2192890

[eph70183-bib-0069] Kiens, B. , Essen‐Gustavsson, B. , Christensen, N. J. , & Saltin, B. (1993). Skeletal muscle substrate utilization during submaximal exercise in man: Effect of endurance training. The Journal of Physiology, 469(1), 459–478. 8271208 10.1113/jphysiol.1993.sp019823 PMC1143880

[eph70183-bib-0070] Klausen, K. , Secher, N. H. , Clausen, J. P. , Hartling, O. , & Trap‐Jensen, J. (1982). Central and regional circulatory adaptations to one‐leg training. Journal of Applied Physiology: Respiratory, Environmental and Exercise Physiology, 52, 976–983. 7085432 10.1152/jappl.1982.52.4.976

[eph70183-bib-0071] Korzeniewski, B. , & Rossiter, H. B. (2020). Exceeding a “critical” muscle P(i): Implications for V˙O2 and metabolite slow components, muscle fatigue and the power‐duration relationship. European Journal of Applied Physiology, 120(7), 1609–1619. 32435984 10.1007/s00421-020-04388-4

[eph70183-bib-0072] Layec, G. , Haseler, L. J. , Hoff, J. , Hart, C. R. , Liu, X. , Le Fur, Y. , Jeong, E. K. , & Richardson, R. S. (2013). Short‐term training alters the control of mitochondrial respiration rate before maximal oxidative ATP synthesis. Acta Physiologica, 208(4), 376–386. 23582030 10.1111/apha.12103 PMC3725772

[eph70183-bib-0073] Leblanc, P. J. , Howarth, K. R. , Gibala, M. J. , & Heigenhauser, G. J. (2004). Effects of 7 week of endurance training on human skeletal muscle metabolism during submaximal exercise. Journal of Applied Physiology, 97(6), 2148–2153. 15220302 10.1152/japplphysiol.00517.2004

[eph70183-bib-0074] LeJemtel, T. H. , Maskin, C. S. , Lucido, D. , & Chadwick, B. J. (1986). Failure to augment maximal limb blood flow in response to one‐leg versus two‐leg exercise in patients with severe heart failure. Circulation, 74(2), 245–251. 3731416 10.1161/01.cir.74.2.245

[eph70183-bib-0075] Liu, Y. , Christensen, P. M. , Hellsten, Y. , & Gliemann, L. (2022). Effects of exercise training intensity and duration on skeletal muscle capillarization in healthy subjects: A meta‐analysis. Medicine and Science in Sports and Exercise, 54(10), 1714–1728. 35522254 10.1249/MSS.0000000000002955

[eph70183-bib-0076] MacInnis, M. J. , Morris, N. , Sonne, M. W. , Zuniga, A. F. , Keir, P. J. , Potvin, J. R. , & Gibala, M. J. (2017). Physiological responses to incremental, interval, and continuous counterweighted single‐leg and double‐leg cycling at the same relative intensities. European Journal of Applied Physiology, 117(7), 1423–1435. 28497384 10.1007/s00421-017-3635-8

[eph70183-bib-0077] MacInnis, M. J. , Skelly, L. E. , & Gibala, M. J. (2019). CrossTalk proposal: Exercise training intensity is more important than volume to promote increases in human skeletal muscle mitochondrial content. The Journal of Physiology, 597(16), 4111–4113. 31309577 10.1113/JP277633

[eph70183-bib-0078] Magnusson, G. , Gordon, A. , Kaijser, L. , Sylvén, C. , Isberg, B. , Karpakka, J. , & Saltin, B. (1996). High intensity knee extensor training, in patients with chronic heart failure. Major skeletal muscle improvement. European Heart Journal, 17(7), 1048–1055. 8809523 10.1093/oxfordjournals.eurheartj.a015001

[eph70183-bib-0079] McDougall, R. M. , Tripp, T. R. , Frankish, B. P. , Doyle‐Baker, P. K. , Lun, V. , Wiley, J. P. , Aboodarda, S. J. , & MacInnis, M. J. (2023). The influence of skeletal muscle mitochondria and sex on critical torque and performance fatiguability in humans. The Journal of Physiology, 601(23), 5295–5316. 37902588 10.1113/JP284958

[eph70183-bib-0080] McGee, S. L. , & Hargreaves, M. (2020). Exercise adaptations: Molecular mechanisms and potential targets for therapeutic benefit. Nature Reviews Endocrinology, 16(9), 495–505. 10.1038/s41574-020-0377-1 32632275

[eph70183-bib-0081] Mitchell, E. A. , Martin, N. R. W. , Bailey, S. J. , & Ferguson, R. A. (2018). Critical power is positively related to skeletal muscle capillarity and type I muscle fibers in endurance‐trained individuals. Journal of Applied Physiology, 125(3), 737–745. 29878875 10.1152/japplphysiol.01126.2017

[eph70183-bib-0082] Molé, P. A. , Oscai, L. B. , & Holloszy, J. O. (1971). Adaptation of muscle to exercise. Increase in levels of palmityl Coa synthetase, carnitine palmityltransferase, and palmityl Coa dehydrogenase, and in the capacity to oxidize fatty acids. Journal of Clinical Investigation, 50, 2323–2330. 5096516 10.1172/JCI106730 PMC292174

[eph70183-bib-0083] Mølmen, K. S. , Almquist, N. W. , & Skattebo, Ø. (2025). Effects of exercise training on mitochondrial and capillary growth in human skeletal muscle: A systematic review and meta‐regression. Sports Medicine, 55, 115–144. 39390310 10.1007/s40279-024-02120-2 PMC11787188

[eph70183-bib-0084] Mølmen, K. S. , Hallén, J. , & Rud, B. (2019). Peripheral adaptations to endurance training—Effect of active muscle mass. Translational Sports Medicine, 2, 240–247.

[eph70183-bib-0085] Mortensen, S. P. , Dawson, E. A. , Yoshiga, C. C. , Dalsgaard, M. K. , Damsgaard, R. , Secher, N. H. , & González‐Alonso, J. (2005). Limitations to systemic and locomotor limb muscle oxygen delivery and uptake during maximal exercise in humans. The Journal of Physiology, 566(1), 273–285. 15860533 10.1113/jphysiol.2005.086025 PMC1464731

[eph70183-bib-0086] Mortensen, S. P. , Egginton, S. , Madsen, M. , Hansen, J. B. , Munch, G. D. W. , Iepsen, U. W. , Åkerström, T. , Pedersen, B. K. , & Hellsten, Y. (2017). Alpha adrenergic receptor blockade increases capillarization and fractional O(2) extraction and lowers blood flow in contracting human skeletal muscle. Acta Physiologica, 221(1), 32–43. 28199786 10.1111/apha.12857

[eph70183-bib-0087] Norman, B. , Sollevi, A. , & Jansson, E. (1988). Increased IMP content in glycogen‐depleted muscle fibres during submaximal exercise in man. Acta Physiologica Scandinavica, 133(1), 97–100. 3227908 10.1111/j.1748-1716.1988.tb08385.x

[eph70183-bib-0088] Olfert, I. M. , Baum, O. , Hellsten, Y. , & Egginton, S. (2016). Advances and challenges in skeletal muscle angiogenesis. American Journal of Physiology‐Heart and Circulatory Physiology, 310(3), H326–H336. 26608338 10.1152/ajpheart.00635.2015 PMC4796623

[eph70183-bib-0089] Ørtenblad, N. , Westerblad, H. , & Nielsen, J. (2013). Muscle glycogen stores and fatigue. The Journal of Physiology, 591(18), 4405–4413. 23652590 10.1113/jphysiol.2013.251629 PMC3784189

[eph70183-bib-0090] Petrova, T. V. , Makinen, T. , & Alitalo, K. (1999). Signaling via vascular endothelial growth factor receptors. Experimental Cell Research, 253(1), 117–130. 10579917 10.1006/excr.1999.4707

[eph70183-bib-0091] Phillips, S. M. , Green, H. J. , Tarnopolsky, M. A. , Heigenhauser, G. J. , & Grant, S. M. (1996). Progressive effect of endurance training on metabolic adaptations in working skeletal muscle. American Journal of Physiology, 270, E265–272. 8779948 10.1152/ajpendo.1996.270.2.E265

[eph70183-bib-0092] Pilotto, A. M. , Adami, A. , Mazzolari, R. , Brocca, L. , Crea, E. , Zuccarelli, L. , Pellegrino, M. A. , Bottinelli, R. , Grassi, B. , Rossiter, H. B. , & Porcelli, S. (2022). Near‐infrared spectroscopy estimation of combined skeletal muscle oxidative capacity and O_2_ diffusion capacity in humans. The Journal of Physiology, 600(18), 4153–4168. 35930524 10.1113/JP283267 PMC9481735

[eph70183-bib-0093] Poole, D. C. , & Musch, T. I. (2023). Capillary‐mitochondrial oxygen transport in muscle: Paradigm shifts. Function, 4(3), zqad013. 37168497 10.1093/function/zqad013 PMC10165549

[eph70183-bib-0094] Poole, D. C. , Musch, T. I. , & Colburn, T. D. (2022). Oxygen flux from capillary to mitochondria: Integration of contemporary discoveries. European Journal of Applied Physiology, 122(1), 7–28. 34940908 10.1007/s00421-021-04854-7 PMC8890444

[eph70183-bib-0095] Poole, D. C. , Ward, S. A. , & Whipp, B. J. (1990). The effects of training on the metabolic and respiratory profile of high‐intensity cycle ergometer exercise. European Journal of Applied Physiology and Occupational Physiology, 59(6), 421–429. 2303047 10.1007/BF02388623

[eph70183-bib-0096] Richardson, R. S. , Poole, D. C. , Knight, D. R. , Kurdak, S. S. , Hogan, M. C. , Grassi, B. , Johnson, E. C. , Kendrick, K. F. , Erickson, B. K. , & Wagner, P. D. (1993). High muscle blood flow in man: Is maximal O2 extraction compromised? Journal of Applied Physiology, 75(4), 1911–1916. 8282650 10.1152/jappl.1993.75.4.1911

[eph70183-bib-0097] Richardson, R. S. , & Saltin, B. (1998). Human muscle blood flow and metabolism studied in the isolated quadriceps muscles. Medicine and Science in Sports and Exercise, 30(1), 28–33. 9475641 10.1097/00005768-199801000-00005

[eph70183-bib-0098] Rosenblat, M. A. , Granata, C. , & Thomas, S. G. (2022). Effect of interval training on the factors influencing maximal oxygen consumption: A systematic review and meta‐analysis. Sports Medicine, 52(6), 1329–1352. 35041180 10.1007/s40279-021-01624-5

[eph70183-bib-0099] Rossman, M. J. , Venturelli, M. , McDaniel, J. , Amann, M. , & Richardson, R. S. (2012). Muscle mass and peripheral fatigue: A potential role for afferent feedback? Acta Physiologica, 206(4), 242–250. 22762286 10.1111/j.1748-1716.2012.02471.x PMC3485418

[eph70183-bib-0100] Rowell, L. B. (1988). Muscle blood flow in humans: How high can it go? Medicine and Science in Sports and Exercise, 20(Sup 1), S97–S103. 3057322 10.1249/00005768-198810001-00001

[eph70183-bib-0101] Rud, B. , Foss, O. , Krustrup, P. , Secher, N. H. , & Hallén, J. (2012). One‐legged endurance training: Leg blood flow and oxygen extraction during cycling exercise. Acta Physiologica, 205(1), 177–185. 22059600 10.1111/j.1748-1716.2011.02383.x

[eph70183-bib-0102] Saltin, B. (1988). Capacity of blood flow delivery to exercising skeletal muscle in humans. American Journal of Cardiology, 62(8), 30E–35E. 10.1016/s0002-9149(88)80007-9 3414535

[eph70183-bib-0103] Saltin, B. , Kiens, B. , Savard, G. , & Pedersen, P. K. (1986). Role of hemoglobin and capillarization for oxygen delivery and extraction in muscular exercise. Acta Physiologica Scandinavica Supplementum, 556, 21–32. 3471054

[eph70183-bib-0104] Savard, G. K. , Richter, E. A. , Strange, S. , Kiens, B. , Christensen, N. J. , & Saltin, B. (1989). Norepinephrine spillover from skeletal muscle during exercise in humans: Role of muscle mass. American Journal of Physiology, 257, H1812–H1818. 2603969 10.1152/ajpheart.1989.257.6.H1812

[eph70183-bib-0105] Sheel, A. W. , Derchak, P. A. , Pegelow, D. F. , & Dempsey, J. A. (2002). Threshold effects of respiratory muscle work on limb vascular resistance. American Journal of Physiology‐Heart and Circulatory Physiology, 282(5), H1732–H1738. 11959638 10.1152/ajpheart.00798.2001

[eph70183-bib-0106] Sidhu, S. K. , Weavil, J. C. , Mangum, T. S. , Jessop, J. E. , Richardson, R. S. , Morgan, D. E. , & Amann, M. (2017). Group III/IV locomotor muscle afferents alter motor cortical and corticospinal excitability and promote central fatigue during cycling exercise. Clinical Neurophysiology, 128(1), 44–55. 27866119 10.1016/j.clinph.2016.10.008 PMC5240388

[eph70183-bib-0107] Skattebo, Ø. , Calbet, J. A. L. , Rud, B. , Capelli, C. , & Hallén, J. (2020a). Contribution of oxygen extraction fraction to maximal oxygen uptake in healthy young men. Acta Physiologica, 230(2), e13486. 32365270 10.1111/apha.13486 PMC7540168

[eph70183-bib-0108] Skattebo, Ø. , Capelli, C. , Rud, B. , Auensen, M. , Calbet, J. A. L. , & Hallén, J. (2020b). Increased oxygen extraction and mitochondrial protein expression after small muscle mass endurance training. Scandinavian Journal of Medicine, & Science in Sports, 30(9), 1615–1631. 32403173 10.1111/sms.13707

[eph70183-bib-0109] Skattebo, Ø. , Peci, D. , Clauss, M. , Johansen, E. I. , & Jensen, J. (2022). Increased mass‐specific maximal fat oxidation rate with small versus large muscle mass exercise. Medicine and Science in Sports and Exercise, 54(6), 974–983. 35576134 10.1249/MSS.0000000000002864

[eph70183-bib-0110] Sundberg, C. W. , & Fitts, R. H. (2019). Bioenergetic basis of skeletal muscle fatigue. Current Opinion in Physiology, 10, 118–127. 31342000 10.1016/j.cophys.2019.05.004 PMC6656370

[eph70183-bib-0111] Thomas, K. , Goodall, S. , & Howatson, G. (2018). Performance fatigability is not regulated to A peripheral critical threshold. Exercise and Sport Sciences Reviews, 46(4), 240–246. 30001270 10.1249/JES.0000000000000162

[eph70183-bib-0112] Tuttle, J. L. , Nachreiner, R. D. , Bhuller, A. S. , Condict, K. W. , Connors, B. A. , Herring, B. P. , Dalsing, M. C. , & Unthank, J. L. (2001). Shear level influences resistance artery remodeling: Wall dimensions, cell density, and eNOS expression. American Journal of Physiology‐Heart and Circulatory Physiology, 281(3), H1380–H1389. 11514310 10.1152/ajpheart.2001.281.3.H1380

[eph70183-bib-0113] Tyni‐Lenné, R. , Gordon, A. , Jensen‐Urstad, M. , Dencker, K. , Jansson, E. , & Sylvén, C. (1999). Aerobic training involving a minor muscle mass shows greater efficiency than training involving a major muscle mass in chronic heart failure patients. Journal of Cardiac Failure, 5(4), 300–307. 10634671 10.1016/s1071-9164(99)91334-9

[eph70183-bib-0114] Volianitis, S. , & Secher, N. H. (2016). Cardiovascular control during whole body exercise. Journal of Applied Physiology, 121(2), 376–390. 27311439 10.1152/japplphysiol.00674.2015 PMC5007320

[eph70183-bib-0115] Wagner, P. D. (1996). Determinants of maximal oxygen transport and utilization. Annual Review of Physiology, 58(1), 21–50. 10.1146/annurev.ph.58.030196.000321 8815793

[eph70183-bib-0116] Weavil, J. C. , Hureau, T. J. , Thurston, T. S. , Sidhu, S. K. , Garten, R. S. , Nelson, A. D. , McNeil, C. J. , Richardson, R. S. , & Amann, M. (2018). Impact of age on the development of fatigue during large and small muscle mass exercise. American Journal of Physiology‐Regulatory, Integrative and Comparative Physiology, 315(4), R741–R750. 29995457 10.1152/ajpregu.00156.2018 PMC6230894

[eph70183-bib-0117] Weavil, J. C. , Thurston, T. S. , Hureau, T. J. , Gifford, J. R. , Aminizadeh, S. , Wan, H. Y. , Jenkinson, R. H. , & Amann, M. (2022). Impact of aging on the work of breathing during exercise in healthy men. Journal of Applied Physiology, 132(3), 689–698. 35085030 10.1152/japplphysiol.00443.2021 PMC8896992

[eph70183-bib-0118] Wetter, T. J. , Harms, C. A. , Nelson, W. B. , Pegelow, D. F. , & Dempsey, J. A. (1999). Influence of respiratory muscle work on VO_2_ and leg blood flow during submaximal exercise. Journal of Applied Physiology (1985), 87(2), 643–651. 10.1152/jappl.1999.87.2.643 10444624

[eph70183-bib-0119] Wilson, D. F. (1994). Factors affecting the rate and energetics of mitochondrial oxidative phosphorylation. Medicine, & Science in Sports, & Exercise, 26, (Supplement), S37. 8133736

[eph70183-bib-0120] Wilson, D. F. , & Erecińska, M. (1985). Effect of oxygen concentration on cellular metabolism. Chest, 88(4), 229S–232S. 4042728 10.1378/chest.88.4_supplement.229s

[eph70183-bib-0121] Zhang, J. , Iannetta, D. , Alzeeby, M. , MacInnis, M. J. , & Aboodarda, S. J. (2021). Exercising muscle mass influences neuromuscular, cardiorespiratory, and perceptual responses during and following ramp‐incremental cycling to task failure. American Journal of Physiology‐Regulatory, Integrative and Comparative Physiology, 321(2), R238–R249. 34189949 10.1152/ajpregu.00286.2020

[eph70183-bib-0122] Zhang, J. , Murias, J. M. , MacInnis, M. J. , Aboodarda, S. J. , & Iannetta, D. (2024). Performance and perceived fatigability across the intensity spectrum: Role of muscle mass during cycling. American Journal of Physiology‐Regulatory, Integrative and Comparative Physiology, 326(6), R472–R483. 38557152 10.1152/ajpregu.00272.2023

[eph70183-bib-0123] Zhang, J. , Pearson, A. Z. , Grunau, M. , Iannetta, D. , Millet, G. Y. , & Aboodarda, S. J. (2025). The role of muscle mass in endurance performance and neuromuscular fatigue: A systematic review and meta‐analysis. Sports Medicine, 55(11), 2809–2824. 40742651 10.1007/s40279-025-02290-7

